# In-house protocol: spin-based viral RNA purification

**DOI:** 10.1186/s13568-022-01413-x

**Published:** 2022-06-09

**Authors:** Mahmoud M. Abdelfattah, Ahmed M. Osman, Mohamed A. Elnagar, Mohamed F. Ibrahim, Magdy Albert, Maya M.Talal, Nasra F. Abdel Fattah, Samah A. Loutfy, Reham Helwa

**Affiliations:** 1 Molecular Cancer Biology Group, Zoology Department, Faculty of Science, Ain Shams University, Cairo, 11566 Egypt; 2 Virology and Immunology Unit, Cancer Biology Department, National Cancer Institute, Cairo University, Cairo, Egypt; 3 Biotechnology Program, Faculty of Science, Ain Shams University, Cairo, Egypt

**Keywords:** Viral RNA, In-house protocol, RNA purification, LPA carrier, Vero cell line

## Abstract

A worldwide shortage of molecular biology consumables is in surge. This includes filter tips, nucleic acid purification kits, polymerases, reverse-transcriptase, and different types of reagents which are included in viral diagnostic kits. In developing countries, the problem is even worse, since there is few capital enterprise to adopt this kind of industry. So, our aim is to develop a suitable, functional, comparable to commercial ones, and affordable in-house protocol to purify viral RNA. We sought some published and commercial RNA purification solutions to set-up an in-house protocol for viral RNA extraction. Solution was prepared accordingly. Also, LPA (linearized polyacrylamide) carrier was evaluated. The whole setting of in-house solutions with addition of LPA carrier was compared to QIAamp viral RNA minikit solutions. Our results showed that linearized polyacrylamide (LPA) carrier in homemade solutions is comparable to poly A carrier which is used in the most commercial kit. In addition, the whole setting of RNA purification solutions did achieve the purpose of viral RNA purification. Also, the result was confirmed using sputum of a Sars-Cov2 infected patient. Our experiments did end up with an affordable homemade solutions for viral RNA purification.

## Introduction

According to Worldmeters data, more than 200 million people were infected with SARS-CoV2 worldwide with 4.4 million death cases (Worldometers 2020). To prevent further infections and control the disease, diagnosis is imperative (Hozhabri et al. 2020; Perrella et al. 2020; Sani et al. 2020). Detecting the viral genome using RT-PCR remains the most sensitive/specific early diagnostic testing comparable to all of other clinical testing which are consequence of disease progression (Azzi et al. 2020; Ponce-Rojas et al. 2021; WHO 2019). Accordingly, billions of testing were used to overcome further transmission of the disease and consequently the need of synthesizing more reagents is raised to cope the pandemic worldwide.

Column-based purification is a type of solid-phase nucleic acid extraction method/protocol which is common in most of the commercial kit in the market (Karl-Heinz Esser et al. 2005). Silica-matrix is commonly used in the nucleic acid purification due to its selective binding to DNA/RNA due to the positive charge of silica with high affinity to nucleic acid (Vikas W. Padhye et al. August 1997). Also, silica-based column purification facilitate fast extractions with good yields of high-quality DNA/RNA (Nicosia et al. 2010). Even though, purification kits with silica-columns are not always affordable due to their high costs especially in non-developed world.

Currently, molecular biologists everywhere are facing shortage of research materials, since several biotechnology products were directed to be utilized in the COVID-19 pandemic. So, our main objective was to construct an affordable in-house protocol to purify viral RNA to execute our research experiment and overcome shortage of lab consumables. Also, comparing the homemade reagents to the commercially available kits was another aim to address in the present study.

## Materials and methods

### Viral RNA purification buffers and LPA carrier

Our target was to prepare solutions similar or compatible to Qiagen formula. The Lysis buffer of Qiagen kit is proprietary and hence it is unavailable for researchers to have same buffer composition. So, the Lysis buffer was prepared with some modifications from previous work (Scallan et al. 2020); 4 M guanidinium thiocyanate (GITC), 55 mM Tris–HCl pH7.5, 25 mM EDTA, and 3% (v/v)Triton X-100. The Lysis buffer is then incubated at 60–70 °C for 15–20 min to dissolve the GITC.

The two washing buffers were prepared similar to RW1 and RPE buffers in Qiagen kit (from US Patent Application US 2011/0221149 A1, Sept. 15, 2011). Washing buffer 1 is (20% Ethanol, 1 M GITC, and 10 mM Tris–HCl pH 7.5) and washing buffer 2 is (80% Ethanol, 100 mM NaCl, and 10 mM Tris–HCl pH 7.5) (Himmelreich and Werner, 2011).

One of our purposes is to prepare inexpensive simple buffers. So, we made linearized polyacrylamide (LPA) carrier and it was prepared as Gaillard and Straus protocol which is approximately one hour experiment (Gaillard and Strauss 1990). The LPA carrier was then stored at −20 $^\circ$C for long-term storage or at 4 $^\circ$C for a month.

EZ-10 spin columns (Cat.#: SD5005) was purchased from biobasic, Canada.

### Samples and RNA purification

In the present study, Vero cells infected with measles virus (Priorix, Germany) was used to optimize RNA purification protocol. Vero cell line is ATCC cell line (cat# CCL-81) and was obtained through a distributing company for biological products & vaccines (VACSERA), Cairo. The cells were grown in DMEM with 10% FBS and 100 IU/ml pencillin/streptomycin. Cells were propagated as a monolayer culture at 37 °C under humidified atmosphere of 5% CO_2_.

Vero cells is permissive for cultivation of measles virus and suitable for viral isolation and replication. Cytopathic effect (CPE) of Measles virus infection into vero cells is characterized by formation of syncytium with spikes as it causes cell fusion together and make cells appeared like giant.

Therefore, vero cells is used for many applications for measles virus like: laboratory diagnosis of measles virus, viral titration to identify TCID50 using Reed & Maunch equation and in antiviral assays (Grigorov et al. 2011; Vaidya et al. 2014). In brief, we used measles vaccine (live attenuated virus) and infect vero cells for 1 h at 37 °C. The experiment was done in three independent repeats.

**Fig. 1:**
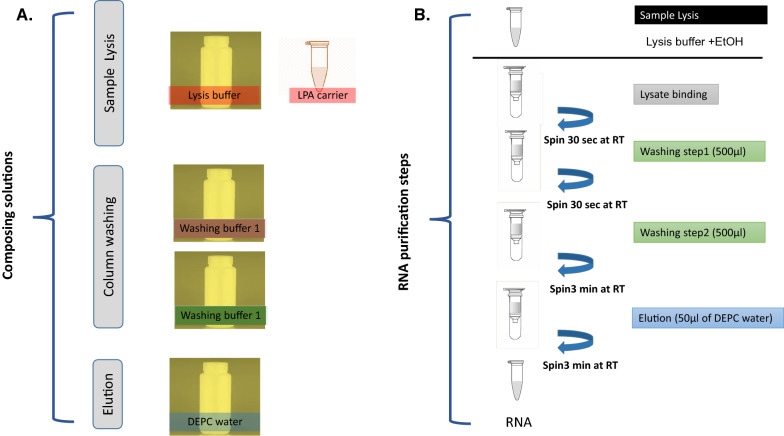
Experiment flow chart of viral RNA purification. **A** kit solutions. **B** procedure summary

To assess the extraction procedure of the viral RNA, RT-PCR was executed for nucleoprotein (N-450) using MeV210 (GCT ATG CCA TGG GAG TRG GAG TGG) and MeV217 (CAA TGA TGG AGG GTA GG) primers previously published (Bankamp et al. 2013). The two primers will amplify nucleotide 1108 to 1734 with 626 bp amplicon. Qiagen QIAamp viral RNA minikit was used as a reference for comparison. A schematic workflow diagram for the whole procedure is represented in Fig. 1.

To validate LPA carrier, the experiment was carried out using Qiagen QIAamp viral RNA minikit (Hilden, Germany). Qiagen poly A carrier was substituted by LPA carrier and all Qiagen solutions (Lysis and washing buffers) were kept to assess whether changing the carrier is influencing the PCR amplification or not. This experiment was carried out in parallel with Qiagen poly A carrier.

From our results, we found that LPA carrier is compatible with Qiagen solutions. Also, the performance of our carrier was comparable to Qiagen carrier. Then, we did proceed to test our solutions with LPA carrier. We did the same as Qiagen protocol as the following with very small modifications:

First, adding the LPA carrier to the Lysis buffer contains was prepared by the following equation which was stated in Qiagen handbook:

$${\text{n}} \times 0.56\,{\text{ml}} = {\text{y}}\,{\text{ml}}$$

$${\text{y}}\,{\text{ml}} \times 10\,\mu {\text{l/ml}} = {\text{z}}\,\mu {\text{l}}$$

Where, n = number of the processed samples. y = calculated volume of lysis buffer. z = volume of carrier RNA–Elution buffer added to lysis buffer

Then the solution was mixed gently by inverting it up and down for 10 times.

- 560 μl prepared lysis buffer containing carrier RNA was added into a 1.5 ml micro-centrifuge tube.
- 140 μl of the sample (in our case, the resuspended cells in PBS) was added to the lysis buffer + LPA carrier in microcentrifuge tube and mixed by pulse-vortexing for 15 s.
- The mixture was incubated at room temperature for 10 min.
- It was centrifuged briefly to remove drops from the inside of the lid.
- 560 μl Ethanol (96–100%) was added to the sample, and mixed by pulse-vortexing for 15 s, followed by brief centrifugation to remove drops from inside the lid.
- Up to 700 μl of the solution from step 6 was applied to spin column (in a 2 ml collection tube), followed by centrifugation at 8000×*g* for 1 min. The collection tube was replaced, the tube containing the filtrate was discarded.
- Step 8 was repeated.
- 500 μl of wash I was added to the spin column. Then was centrifuged at 8000×*g* for 1 min. The flow-through was discarded.
- 500 μl of wash II was added. Then it was centrifuged at full speed (14,000×*g*) for 3 min.
- Spin column was transferred in a new 2 ml collection tube, the old collection tube was discarded with the filtrate and was centrifuged at full speed for 1 min.
- Spin column was placed in 1.5 ml micro-centrifuge tube, 50 μl of Elution buffer was added, was incubated at room temperature for 1 min.
- The column was centrifuged at 8000×*g* for 1 min.
- Another 50 µl of elution was added and then was centrifuged at 8000×*g* for a min.

Every experiment with our solution was done in comparison to Qiagen QIAamp viral RNA minikit. For further validation, before RNA purification, Vero cells were diluted to 1:1, 1:10, 1:100, and 1:1000 and one-step RT-PCR was performed to check the sensitivity of the in-house protocol using Qiagen OneStep RT-PCR kit (Qiagen, Hilden, Germany). The thermal profile was 30 min at 50 °C, 15 min at 95 °C, forty cycles of amplification (95 °C for 30 s, 55 °C for 30 s, and 72 °C for 30 s), and final extension at 72 °C for 10 min.To visualize PCR products, 2% agarose gel electrophoresis was performed. 50 bp Generuler DNA ladder (Thermoscientific) was used as a standard. The electrophoresis pictures were analyzed using ImageLab software version 6.1 (Biorad).

### Covid-19 RNA purification and amplification from a sputum sample

A sputum sample was collected from a volunteer with positive coronavirus. RNA extraction was performed using QIAamp viral RNA extraction kit in comparison to our in-house solutions. The purified RNA concentration was measured using Nanodrop spectophotometer. The viral RNA was detected by real-time PCR assay using Vitro S.A One-Step RT Kit. Standard curves were constructed using Serial dilutions from 10^6^ copies/reaction to 10^1^ copies/reaction of synthetic fragments of the N gene in the FAM channel and E gene in the ROX channel and the both genes were detected at 10 copies/reaction.

## Results

### Validation workflow

**Fig. 2:**
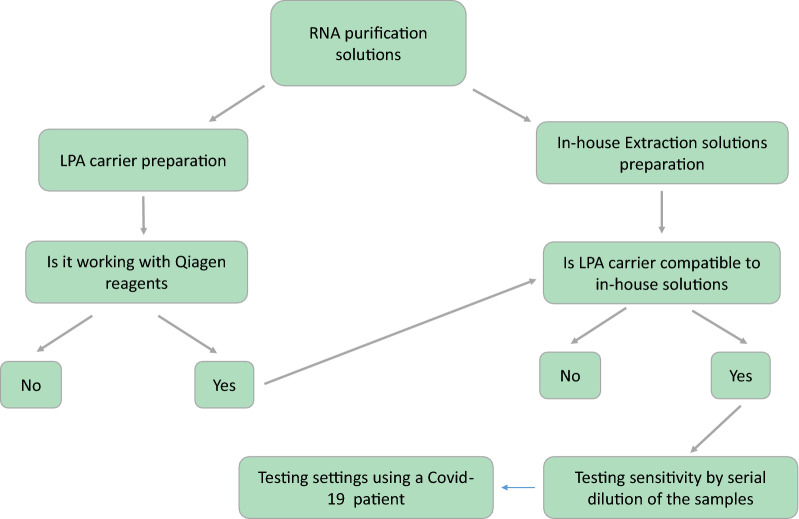
The validation workflow of the in-house protocol

According to our objective, the solutions were prepared and tested to execute the RNA purification experiment in the cheapest format, but at the same time with quality comparable to commercially available kits. So, firstly, LPA carrier was tested with QIAamp viral RNA mini kit. Then, the experiments were executed in a workflow as shown in Fig. 2.

### Using LPA carrier is comparable to the commercial poly A included in QIAamp viral RNA mini kit

**Fig. 3:**
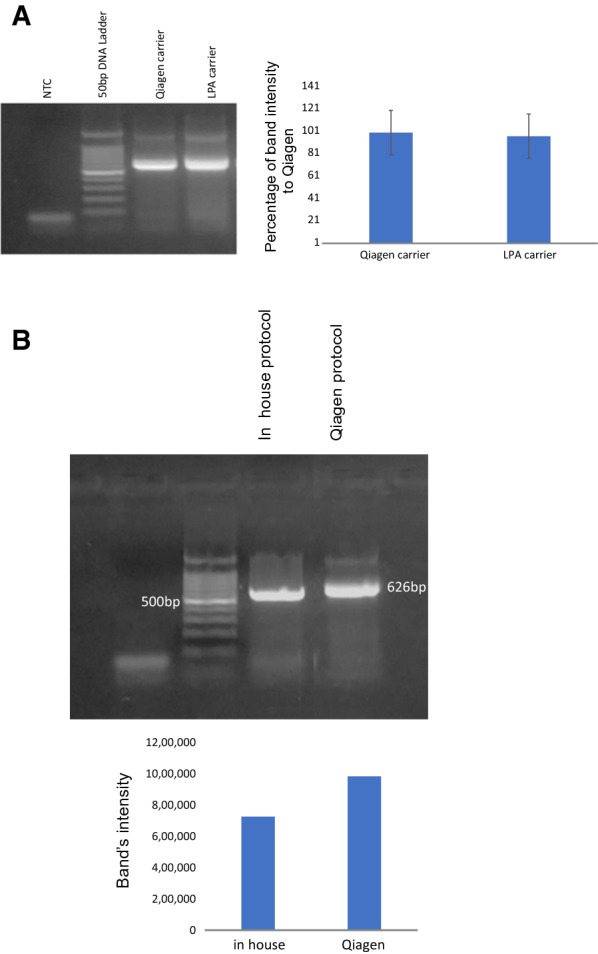
Evaluation of LPA carrier versus Qiagen carrier. RNA was purified from infected Vero cells with measles virus. The same amount of cells used as startup point to compare Qiagen carrier and LPA carrier using all Qiagen reagents (i.e. the only difference between the tubes is type of carrier). **A** RT-PCR of N-450 using MeV210 and MeV217 primers for RNA purified with Qiagen reagents and LPA carrier. The samples were compared to whole Qiagen setting control with their carrier by running on 2%Agarose with histogram showing percentage of bands intensities comparable to Qiagen kit. **B** RT-PCR of N-450 using MeV210 and MeV217 primers for RNA purified with in-house protocol comparable to Qiagen QIAamp viral RNA mini kit

The main expensive components in RNA purification kit are the carrier and spin-columns. Therefore, in order to make in-house affordable protocol, we did synthesize linearized polyacrylamide carrier in our lab. To test efficiency of the LPA carrier, solutions of QIAamp viral RNA mini kit were used and the poly A carrier was substituted with LPA carrier. Our control was QIAamp viral RNA mini kit whole reagents. After the purification, one-step RT-PCR for measles virus was performed to assess the RNA purification process. Then, amplification of N-450 of Measles virus was visualized by gel electrophoresis and bands intensities were measured using ImageLab software version 6.1 (Biorad) (Fig. 3). According to this experiment results, both carriers were reasonable to use, the amplicon intensity obtained using LPA carrier in RNA purification was 98% of Qiagen QIAamp viral RNA mini kit as shown in Fig. 3. From this experiment, two observations were very useful for the next step of validation: (1) LPA carrier could be used for viral RNA purification, and (2) LPA carrier is compatible to solutions of QIAamp viral RNA mini kit.

### The in-house solutions did successfully purify viral RNA

Following step of validation was to test LPA carrier with the homemade solutions. Hence, we did follow the protocol as written in the materials and methods section. The RNA purification of measles virus was tested using one-step N-450 and MeV210 and MeV217 primers RT-PCR, and then visualized by 2% gel electrophoresis. Both Qiagen QIAamp viral RNA mini kit (all solutions with their carrier) and in-house solutions were carried out in parallel to control the experiment. The captured pictures of electrophoresis were analyzed using ImageLab software version 6.1 (Biorad).

In comparison to Qiagen QIAamp viral RNA mini kit, band intensity was approximately 80% of commercially available Qiagen kit as shown in Fig. 3, which is still very useful for research labs to have.

**Fig. 4:**
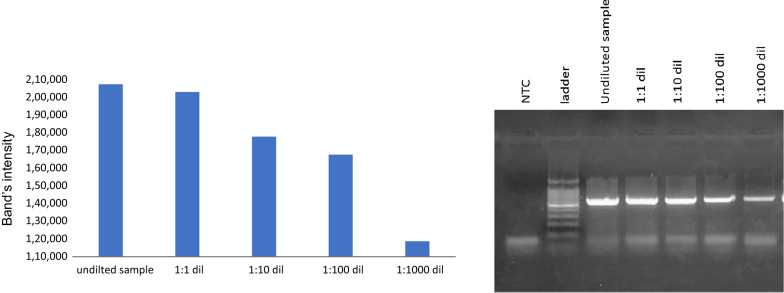
RT-PCR of N-450 using MeV210 and MeV217 primers for RNA purified with our homemade kit solutions and carrier. The RT-PCR was done for RNA extracted after serial dilution of infected Vero cells. Amplicons with 626 bp were detected by 2% agarose gel electrophoresis. 50 bp generuler ladder (thermoscientific) was used as standard

For further sensitivity testing, Vero cells was diluted before RNA purification to 1:1, 1:10, 1:100, and 1:1000. Also, RT-PCR for N-450 was performed for the purified RNA. The PCR amplicon was detected in all samples with different dilution as shown Fig. 4.

### RNA purification was successfully purified and amplified from sputum of covid-19 patient

A sputum sample from covid-19 positive volunteer was subjected to RNA purification using our in-house solutions in comparison to Qiagen kit. The RNA concentration was measured as shown in Table 1. The obtained concentrations were very comparable. Subsequently the RNA was subjected to qRT-PCR. The cycle thresholds and copy numbers are shown in Table 1. According to this result, the in-house solutions have very close results to Qiagen kit.

**Table 1:** RNA purification from a covid-9 positive sputum sample

	Concentration (ng/µl)	Purity (260/280 ratio)
**A** RNA purification data		
QIagen extraction kit	20.9	1.98
In-house solutions	19.8	2.02

	N-gene CT	N-gene viral load Copies/ml	E-gene CT	E-gene viral load Copies/ml	RNaseP CT	Positive/negative
**B** qRT-PCR results						
QIagen extraction kit	20.83	1.6 × 10^5^	23.92	7.13 × 10^3^	26.96	Positive
In-house solutions	21.9	6.9 × 10^4^	24.67	4.16 × 10^3^	27.3	Positive
Positive control	19.4	4.9 × 10^5^	24.52	4.6 × 10^3^	28.59	Positive
Negative control	U.D	U.D	U.D	U.D	U.D	Negative

## Discussion

In SARS-CoV2 pandemic, screening of suspects is highly recommended to reduce the disease transmission (FDA 2020; Hozhabri et al. 2020; WHO 2019). Thus, the pandemic caused severe shortage of clinical and science consumables. Also, funding opportunities for scientific researchers are prioritized to cope the pandemic, rendering most of research labs in real dilemma. Since the need is behind every single experiment in science, we aimed to optimize in-house cheap RNA purification protocol to minimize our research budget and make the conditions easier and feasible for other biologists to continue with their research during the pandemic.

Silica-based RNA purification are the most common in both research and clinical diagnostics labs due to their advantage of efficient saving of time and high purity of the eluted nucleic acid (Nicosia et al. 2010). However, the kits are not affordable for many labs because of the high cost, especially in non-developed countries. Here, we did composed RNA purification solutions which are Lysis buffer, LPA carrier, and washing solutions. Most companies keep the ingredients of the Lysis buffer as a proprietary with unknown concentration of guanidine iso-thyocyanate. To execute our experiment, we did use a recipe from another research group (Scallan et al. 2020). Washing buffers were prepared according to Qiagen released recipe (Himmelreich and Werner, 2011).

To make in-house viral RNA purification buffers for silica-based extraction is to use a type of carrier to increase the RNA yield, especially with those samples having low copy numbers of viruses/genetic materials. Most of the commercially available kits include poly A carrier RNA. However, it is too expensive for the current pandemic situation especially in poor labs. LPA carrier is a very cheap, easy to prepare, and efficient neutral carrier for precipitating picogram amounts of nucleic acids with ethanol and doesn’t interfere with downstream application (Gaillard and Strauss 1990). We substituted poly A carrier in Qiagen QIAamp viral RNA mini kit with LPA carrier and it was working with comparable results to Qiagen carrier.

In COVID-19 pandemic, several kit were compared with different performance as published by Amrosi *et.al*. (Ambrosi et al. 2021). Also, many research groups did optimize methods to purify viral RNA (Page et al. 2022; Zheng et al. 2022). In the present study, infected Vero cells with measles virus were used to test the whole setting of the purification solutions including Lysis buffer, LPA carrier, washing buffers, and spin columns. Then, the purification procedure was evaluated by RT-PCR and every sample was controlled by parallel purification with Qiagen QIAamp viral RNA mini kit. Then, band intensities were compared. Also, to test the sensitivity of our setting, serial dilution of samples were exploited. In addition, our in-house solution with LPA carrier did perform a comparable purification of covid-19 RNA to Qiagen QIAamp viral RNA mini kit. This result was confirmed quantitatively using real-time PCR.

In conclusion, our results are giving alternative homemade solutions to purify viral RNA. The method is affordable alternative for researchers during the crisis of materials shortage in the market and comparable to the commercial kit. Moreover, using LPA carrier is novel in RNA purification which is a big advantage over poly A carrier in the cost and easy preparation. With further future modification, it could be used in diagnostics as well. Also, we are now working in synthesizing spin-column which will make the protocol more affordable in low-income countries.

## Data Availability

The datasets used and/or analyzed during the current study are available from the corresponding author on reasonable request.

## Abbreviations

LPA: Linearized polyacrylamide
RT-PCR: Reverse transcription polymerase chain reaction
qRT-PCR: Quantitative reverse transcription polymerase chain reaction
RNA: Ribonucleic acid
cDNA: Complementary DNA
